# Self-Compassion and Psychological Flourishing Among College Students: The Mediating Role of Hope and the Moderating Role of Emotion Regulation

**DOI:** 10.3390/bs14121149

**Published:** 2024-11-29

**Authors:** Chunying Liu, Pingting Lin, Zhiheng Xiong

**Affiliations:** 1 School of Marxism, Nanjing Normal University of Special Education, Nanjing 210038, China; 260026@njts.edu.cn; 2 School of Special Education, Nanjing Normal University of Special Education, Nanjing 210038, China; lynnpt@njts.edu.cn; 3 School of Humanities, Southeast University, Nanjing 211189, China

**Keywords:** college students, self-compassion, psychological flourishing, hope, emotion regulation

## Abstract

College students face pressure from various aspects such as academics, employment, and interpersonal relationships, and their mental health is receiving increasing attention. This study used a cross-sectional, descriptive correlational design to recruit 842 college students to explore the relationship between self-compassion and psychological flourishing and the underlying psychological mechanisms. With gender added as a control variable, the results showed the following: (1) self-compassion had a positive predictive effect on psychological flourishing in college students; (2) hope partially mediated the predictive effect of self-compassion on psychological flourishing in college students; (3) the first half path of the mediation model was moderated by emotion regulation. In conclusion, this study revealed the underlying mechanisms of the association between self-compassion and psychological flourishing. The mechanisms of increasing college students’ psychological flourishing are the positive impact of hope on the relationship of self-compassion and psychological flourishing, and emotional regulation enhancing the impact of hope. These findings not only enrich the theoretical framework of the relationship between self-compassion and psychological flourishing but also provide practical guidance for future applications of mindfulness and compassion skills to promote physical and mental health. Future research could further explore the effectiveness of self-compassion interventions in different populations, and how cultivating mindfulness and compassion skills can increase individuals’ levels of self-compassion, thereby promoting mental health and overall well-being.

## 1. Introduction

Entering college marks a significant shift in one’s social role, which often brings about pressure and pain concerning lifestyle, social responsibility, living environment, and other related factors [1]. They must make important decisions about their future careers and understand the social responsibilities that they need to accept in the future, leading to increased stress in various aspects including academics, finances, interpersonal relationships, and life planning [2,3]. If these difficulties are not dealt with effectively, mental health issues such as anxiety and depression may occur [4]. With the increasing attention on the mental health of college students, self-compassion as a positive psychological trait has gradually gained prominence in the college population. However, relatively little has been conducted to explore how self-compassion promotes the internal mechanisms of psychological flourishing, especially the mediating role of hope and the moderating role of emotion regulation. The present study aimed to explore in depth the relationship between self-compassion and psychological flourishing among college students from two perspectives: the mediating role of hope and the moderating role of emotion regulation. By constructing a moderated mediation model, we aim to test whether and how self-compassion influences psychological flourishing through hope, and to explore how emotion regulation plays a moderating role in this process. This study contributes to the existing literature in three ways: first, it extends the research on the relationship between self-compassion and psychological flourishing by shedding light on the mediating role of hope, providing a new perspective for understanding how self-compassion contributes to psychological flourishing; second, it delves into the moderating role of emotion regulation in this mediated model, providing a theoretical rationale for the development of effective mental health intervention strategies; and, finally, the model proposed in this study provides practical guidance for reducing psychological distress and enhancing psychological flourishing among college students.

## 2. Literature Review

### 2.1. Self-Compassion and Psychological Flourishing

Self-compassion refers to individuals holding an open and tolerant attitude towards negative events they have experienced, giving an unbiased understanding of the pain and defeat they have encountered, and regarding their experience as a common one shared by all mankind [5]. This helps to reduce their pain by avoiding excessive attention and exaggeration of current negative emotions. Self-compassion is also considered a precious mental resource, viewed as a positive internal power of humanity when confronting life [6]. It could arouse one’s positive qualities such as goodness, openness, and sense of balance, and promote deeper interpersonal relationships [7]. Psychological flourishing, as a psychological trait of an individual, can act as a protective factor that maintains the normal functioning of the individual’s body and mind [8]. It is characterized by meaning in life, self-esteem, optimism, competence, and positive relationships with others [9]. Numerous studies show that psychological flourishing is strongly associated with higher levels of mental health, and satisfaction with basic psychological needs [10,11,12]. Positive psychology theory suggests that a person’s ability to maintain a positive attitude and adopt effective coping strategies is often seen as one of the most important factors influencing the direction of their psychological state [13]. Previous research also has found that higher levels of self-compassion are associated with some positive psychological constructs [14,15]. In contrast, lower levels of self-compassion are associated with depression and anxiety [16,17]. Given the impact of positive psychology on an individual’s optimal state of functioning and well-being, this study draws on the theoretical framework and research methodology of positive psychology to explore the positive aspects of an individual’s psychological resources. Therefore, Hypothesis 1 was proposed: Self-compassion has a positive predictive effect on psychological flourishing in college students.

### 2.2. The Mediating Role of Hope

Hope is an intrinsic factor that motivates individuals positively, and is composed of a reciprocally derived sense of successful agency (goal-directed determination) and pathways (planning of ways to meet goals) [18]. Previous studies show that hope significantly predicts mental health [19,20,21]. Based on the aforementioned, it can be said that hope encompasses one’s self-expectations, assessment of others, and a positive outlook on the future. In everyday life, hope can have a profound effect on an individual’s emotions, behaviors, and mental health. It may mediate the relationship between self-compassion and psychological flourishing among students. On the one hand, there is a strong link between self-compassion and hope. Self-compassion involves perceiving one’s dilemmas and frustrations with a positive emotional response, leading to individuals with high self-compassion more inclined to connect with others and pay more attention to their needs [22].Therefore, individuals with high self-compassion are more likely to face life positively and optimistically. Their goal-directed behaviors are fully stimulated, thus increasing their hope for their future life. On the other hand, hope is also closely associated with psychological flourishing and helps to develop psychological flourishing positively. Individuals with hope tend to believe that they can overcome difficulties, which helps to increase their self-motivation to take action towards goals [23]. Such goal-directed action helps to realize self-worth and a sense of accomplishment among students, which in turn increases psychological flourishing. Self-determination theory also emphasizes people’s pursuit of feeling control and autonomy over their lives [24]. Hope provides the motivation and confidence to achieve their goals, thereby fulfilling the need for self-determination and contributing to psychological flourishing [25,26]. Then, Hypothesis 2 was proposed: Hope significantly mediates the predictive effect of self-compassion on psychological flourishing in college students.

### 2.3. The Moderating Role of Emotion Regulation

The ability to regulate one’s emotions is considered essential for mental health [27]. College students just entering college tend to experience negative emotions due to factors such as adjusting to the new environment and academic pressure [28]. They may feel stressed because of their studies, find life more challenging, and produce self-doubt [29]. In addition, students also hold hope for their future, aspiring to attain good grades, make friends who share their interests, and achieve their goals and dreams at college [30]. Studies showed that emotion regulation is associated with both self-compassion and hope [31,32]. Based on the above, it can be found that the relationship between self-compassion and hope may be moderated by the level of emotion regulation among students. Self-compassion is more strongly related to hope when individuals have higher emotion regulation. This is probably because individuals with higher emotion regulation are better able to adjust their emotional responses in the face of distress and feel self-compassion [33,34]. Positive self-compassion helps individuals reassess their dilemmas and maintain hope for the future, strengthening the connection between self-compassion and hope [35]. Conversely, individuals with low emotion regulation may have difficulty adjusting emotional responses in challenging situations, thus reducing their self-compassion and affecting their hope [36,37]. Therefore, Hypothesis 3 was proposed: Self-compassion significantly predicts hope through the moderating effect of emotion regulation in college students.

### 2.4. The Present Study

**Figure 1:**
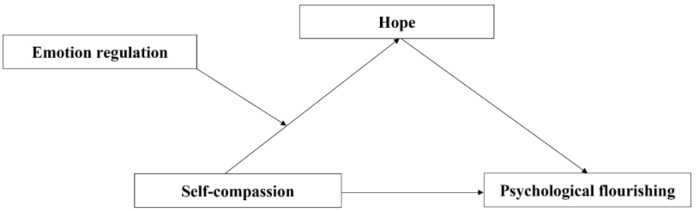
The proposed moderated mediation model.

This study aimed to explore the effects of self-compassion and hope on psychological flourishing in college students, and to analyze the mediating effect of hope and the moderating effect of emotion regulation. Therefore, this study proposed a moderated mediation model (Figure 1).

## 3. Method

### 3.1. Participants

An introductory section was included in the questionnaire to explain the purpose of the study, the rationale for collecting survey data, and the precise instructions for completing the questionnaire. By cluster sampling, we collected data from three universities in Jiangsu and Zhejiang provinces (China). We recruited 960 college students to participate in the survey. After the survey was completed, we reviewed all the data and eliminated questionnaires that were not filled out completely and had the same answer for each question. Thus, 842 valid questionnaires were finally collected, and the effective recovery rate was 87.71%. In total, 154 male college students and 688 female college students were included, with their age ranging from 17 to 21 years old (*M* = 18.51, *SD* = 0.78). Before taking part in the survey, the participants were informed that the study was anonymous and voluntary, with the right to withdraw at any time. This study complies with the Declaration of Helsinki and adheres to ethical norms as approved by the Ethics Committee of Nanjing Normal University of Special Education.

### 3.2. Research Tools

#### 3.2.1. Self-Compassion Scale

The 12-item Self-Compassion Scale-Short Form (SCS-SF) was developed by Raes et al. [38]. A sample item is “I try to be understanding and patient towards those aspects of my personality that I do not like”. Participants indicated responses using a 5-point scale ranging from 1 (*Almost Never*) to 5 (*Almost Always*). Higher scores indicated greater self-compassion. Previous studies have confirmed that the scale is applicable to Chinese culture and has good reliability and validity among college students [39]. In this study, the Cronbach’s α coefficient of this scale was 0.80.

#### 3.2.2. Flourishing Scale

The 8-item Flourishing Scale was used to assess psychological flourishing [9]. A sample item is “I actively contribute to the happiness and well-being of others”. With a 7-point Likert response scale ranging from 1 (*Totally Disagree*) to 7 (*Totally Agree*), higher scores represented higher levels of psychological flourishing. Previous studies have confirmed that the scale is applicable to China and has good reliability and validity among college students [40]. The Cronbach’s α coefficient of the scale in this study was 0.81.

#### 3.2.3. The Hope Scale

Hope was measured by the Hope Scale [41]. The 12-item scale consisted of two subscales including four pathways items (e.g., “I can think of many ways to get out of a jam”), four agency items (e.g., “I energetically pursue my goals”), and four fillers excluded from the total score. Items were rated on a 4-point scale ranging from 1 (*Definitely False*) to 4 (*Definitely True*). Higher scores represented greater hope. Previous studies have confirmed that the scale is applicable to a sample of Chinese students with high reliability and validity [42]. The Cronbach’s α coefficient of the scale in this study was 0.91.

#### 3.2.4. Emotion Regulation Attitude Scale

Emotion regulation was measured by the Emotion Regulation Attitude scale [43]. A sample item is “I think people should be free to express their emotions”. The 9-item scale was measured in a 5-point Likert scale ranging from 1 (*strongly disagree*) to 5 (*strongly agree*). Higher scores indicated better ability to regulate emotions. Previous studies have confirmed that the scale has been applied to a sample of Chinese students with high reliability and validity [44]. The Cronbach’s α coefficient of the subscale was 0.83.

### 3.3. Statistical Analysis

All statistical analyses were carried out using SPSS 26.0 and Process 3.3. SPSS 26.0 was used for data entry, data collection, descriptive statistical analysis, and correlation analysis. After calculating bivariate correlations, Hayes’ PROCESS macro model 4 was used to test the mediating role of hope between self-compassion and psychological flourishing [45]. The moderating effect of emotion regulation on the first path of the mediation process was examined using Hayes’ PROCESS macro model 7 [45]. Before applying models 4 and 7, standardized scores were calculated for all variables, along with the interaction terms calculated from the standardized scores. Bootstrapping was used to test confidence intervals, with a 95% confidence interval (CI) calculated through 5000 repeated samples.

## 4. Results

### 4.1. Common Method Deviation

There may be common method bias when data are collected by participants’ self-report [46]. Therefore, Harman’s single-factor test was used to avoid common method bias. The results show that there were six factors with the root of the measured eigenvalue >1, and the first factor was less than the critical standard, indicating that there was no serious common method deviation in this study.

### 4.2. Descriptive Statistics and Related Analysis for Each Variable

As shown in Table 1, self-compassion, psychological flourishing, hope, and emotion regulation were significantly and positively associated with each other among college students. Gender had a significant, positive correlation with psychological flourishing and emotion regulation, respectively. Therefore, gender was used as a control variable in the subsequent analyses.

### 4.3. Testing for the Mediation Effect

**Figure 2:**
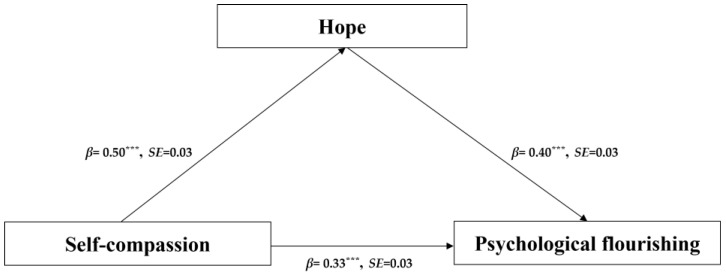
Standardized path coefficients depicting mediation of hope in the association between self-compassion and psychological flourishing (*N* = 842). Note: *** *p* < 0.001.

The mediation effect refers to the relationship between variables; X → Y is an indirect effect through the intermediate variable M, and this indirect causal relationship is called the mediation effect [47]. SPSS Process Model 4 was used to examine whether hope mediated the relationship between self-compassion and psychological flourishing [45]. After controlling for gender, mediating effect analyses were performed (Table 2). The results of the first step showed that self-compassion significantly and positively predicted hope (*β* = 0.50, *p* < 0.001). When the mediating variable was not included, self-compassion significantly and positively predicted psychological flourishing (*β* = 0.52, *p* < 0.001). After including the mediating variable, hope significantly positively predicted psychological flourishing (*β* = 0.40, *p* < 0.001), and self-compassion still significantly and positively predicted psychological flourishing (*β* = 0.33, *p* < 0.001), indicating the mediating effect between self-compassion and psychological flourishing (Figure 2). Indirect effects were further tested using the bias-corrected percentile Bootstrap method. The results showed that hope had an indirect effect on the relationship between self-compassion and psychological flourishing, that was, hope played a partial mediating role, and the mediating effect accounted for 37.74% of the total effect (*β* = 0.20, SE = 0.03, 95% CI [0.15, 0.25]).

### 4.4. Moderated Mediation Effect Analysis

**Figure 3:**
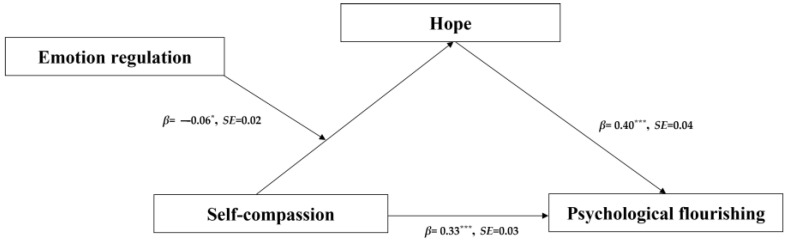
Standardized path coefficients depicting a moderated mediation role of emotion regulation and hope in the association between self-compassion and psychological flourishing (*N* = 842). Note: * *p* < 0.05, *** *p* < 0.001.

The moderation effect refers to the moderating effect of variable U when the size or positive and negative directions of the correlation between variables X and Y are influenced by variable U [47]. Model 7 in SPSS Process was used to examine the moderated effect of emotion regulation between self-compassion and hope [45]. The results after adding control variables (i.e., gender) are shown in Table 3 and Figure 3. The interaction term of self-compassion and emotion regulation had a significant predictive effect on hope (*β* = −0.06, *p* < 0.05). 

**Figure 4:**
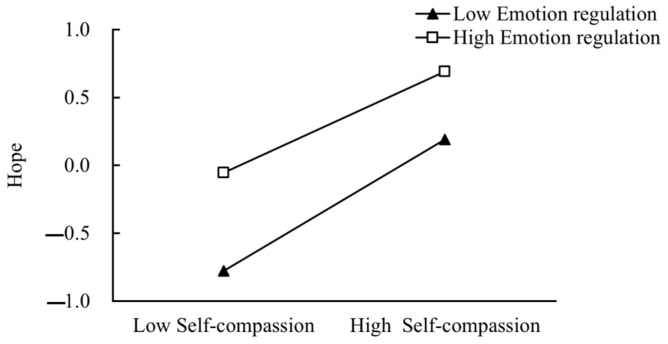
The moderating role of emotion regulation.

To test how the different levels of emotion regulation affected self-compassion on hope, we divided emotion regulation into two levels, “low emotion regulation” (*M* + 1*SD*) and “high emotion regulation” (*M* − 1*SD*), and examined the predictive effect of self-compassion on hope in two separate groups of subjects. There was a significant, positive predictive effect of self-compassion on hope (*β*_simple slope_ = 0.48, *p* < 0.001) among college students with low emotion regulation. For college students with high emotion regulation, self-compassion also significantly positively predicted hope (β_simple slope_ = 0.37, *p* < 0.001), but the effect was weaker (Figure 4).Thus, it is found that emotion regulation has a significant moderating effect between self-compassion and hope.

## 5. Discussion

This study confirms the positive association between self-compassion and psychological flourishing through the survey of a group of college students, verifying the mediating role of hope in this relationship and the moderating role of emotion regulation. This finding provides theoretical support for understanding the internal mechanism of psychological flourishing and is also important for promoting mental health among college students.

### 5.1. The Relationship Between Self-Compassion and Psychological Flourishing

The results showed that self-compassion significantly and positively predicted psychological flourishing in college students. The higher the self-compassion, the higher the psychological flourishing, confirming Hypothesis 1. This was consistent with previous studies that self-compassion, as a positive self-attitude, allowed individuals to treat themselves with more tolerance and understanding in the face of challenges, which in turn improves psychological flourishing [48,49]. This finding also supported Conservation of Resources Theory (COR Theory) and Compassion Focused Therapy (CFT) [50,51]. COR Theory suggests that self-compassion can be viewed as a valuable psychological resource that helps individuals become more resilient and adaptable in the face of challenges. CFT emphasizes the importance of positive psychological effects on an individual’s psychological development. Through self-compassion training, individuals can better focus on the present and develop a sense of inner warmth and security, which effectively improves their mental health. High self-compassion not only helps individuals to cope effectively with stress but also facilitates the use, flexibility, and integration of available external support resources [52]. This study reveals the positive impact of self-compassion on psychological flourishing among college students, providing a new perspective for improving their mental health. To promote psychological flourishing among college students, college teachers can emphasize the importance of self-compassion in mental health education and teach students how to treat themselves with greater compassion and understanding. For college students, mindfulness can help them learn to observe and accept their emotional feelings. This can enhance their ability to be self-compassionate, which in turn promotes the achievement of psychological flourishing.

### 5.2. The Mediating Role of Hope

This study found that hope played a partial mediating role between self-compassion and psychological flourishing in college students, thus supporting Hypothesis 2. Specifically, it deepens our understanding of the mechanisms of self-compassion by translating the positive effects of self-compassion into real-life adaptive behaviors. In addition, the current study explores the intrinsic link between self-compassion and psychological flourishing and provides a plausible explanatory framework for this association. 

Regarding self-compassion and hope, the results were similar to those of previous studies [53,54] and tend to support the idea that self-compassion may be a potential driver of hope. Individuals are able to be more tolerant and accepting of their own shortcomings when self-compassion is cultivated [55]. This self-acceptance and caring gives the individual inner strength so that hope is no longer just an external stimulus, but is transformed into positive beliefs that come from deep within and drive the individual forward [56]. For hope and psychological flourishing, the results showed a significant positive correlation, similar to previous studies [57,58]. When we are hopeful, this positive mindset not only increases our optimism but also our resilience and perseverance in the face of challenges. This positive orientation enables individuals to pursue their dreams with more determination and promotes the generation of psychological flourishing [25]. The results also support the meaning-seeking model, which suggests that people seek fulfillment and meaning in their lives to reduce their exposure to negative external circumstances [59]. When individuals establish clear expectations for the future and a clear path to achieve their goals, they are more likely to generate positive energy within themselves. Hope may play a significant role in this process. In response to the findings of this study, universities can take targeted measures to help college students. For instance, they can provide career planning, various extracurricular activities, and practical opportunities to help college students better understand their strengths and interests. This clarifies their future directions and goals, thereby enhancing their hope. Simultaneously, teachers can utilize multiple teaching methods to establish a constructive academic environment, ignite the curiosity and drive of college students, and arouse their desire to learn. This approach will facilitate improved academic outcomes and foster a greater sense of hope.

### 5.3. The Moderating Role of Emotion Regulation

This study also demonstrated that emotion regulation played a moderating role in the indirect relationship between self-compassion and hope, thus validating Hypothesis 3. This finding supports the expansion-construction theory of positive emotions [13]. The theory emphasizes that adaptive emotion regulation strategies can enhance an individual’s psychological resilience in the face of challenges, thereby facilitating the generation of positive psychological resources. When college students are able to face their emotional experiences with a tolerant attitude, they are more likely to use adaptive emotion regulation strategies to manage those emotions [22]. This approach can help students learn to tolerate their mistakes, thereby reducing the impact of negative emotions and transforming them into a positive outlook with hope for the future. On the contrary, students with weaker emotional regulation may also feel a degree of self-compassion. However, the possible lack of effective emotional coping strategies makes it more difficult to translate this self-compassion into a sense of hope for the future [37,60]. Therefore, college psychology teachers can help students learn effective emotion regulation skills by offering emotion management courses and psychological counseling. For the students themselves, they can actively explore and practice various emotion regulation strategies to improve their emotion regulation skills when facing challenges in their studies or life.

### 5.4. Limitations

This study also has some limitations. The use of cross-sectional data may limit the causal relationship between variables. Future research could use a longitudinal design to clarify the relationship between variables. Additionally, further studies could explore the specific mechanisms of emotion regulation in depth and figure out more effective ways to enhance individuals’ emotion regulation. This could lead to an improvement in the psychological adjustment and quality of life of students.

## 6. Conclusions

In conclusion, the present study makes an important contribution to research in related fields by constructing and validating a moderated mediation model that provides insight into the complex mechanisms linking self-compassion and psychological flourishing. Specifically, our study identifies hope as a mediating variable linking self-compassion and psychological flourishing. In addition, our study reveals the moderating role of emotion regulation in the link between self-compassion and hope. This finding suggests that individuals may be able to enhance the positive impact of self-compassion on hope through effective emotion regulation strategies when faced with challenges and difficulties. These findings not only enrich the theoretical framework of the relationship between self-compassion, hope, emotion regulation, and psychological flourishing, but also provide important practical guidance for promoting psychological health and well-being among college students. By enhancing self-compassion and emotion regulation, individuals are able to increase their sense of hope, which in turn promotes psychological flourishing and higher levels of psychological well-being. Therefore, this study not only represents a theoretical breakthrough, but also has great value and potential for practical application. We expect that these findings will further advance research in related fields and make a greater contribution to improving the mental health of college students.

## Figures and Tables

**Table 1:** Descriptive statistics and correlations among variables (*N* = 842).

Variable	*M*	*SD*	1	2	3	4	5
1. Gender	0.18	0.39	1				
2. Self-compassion	40.86	6.70	−0.03	1			
3. Psychological Flourishing	39.06	6.55	−0.14 **	0.53 **	1		
4. Hope	23.72	3.60	−0.05	0.50 **	0.57 **	1	
5. Emotion regulation	30.87	5.10	−0.11 **	0.22 **	0.45 **	0.41 **	1

Note: gender: 0 = female, 1 = male; *M* is mean value; *SD* is standard deviation. ** *p* < 0.01.

**Table 2:** Testing for the mediation effect (*N* = 842).

Dependent Variable	Independent Variable	*R* ^2^	*F*	*β*	*SE*	*t*
Hope	Gender	0.25	138.35 ***	−0.04	0.03	−1.33
	Self-compassion			0.50	0.03	16.54 ***
Psychological Flourishing	Gender	0.29	173.81 ***	−0.12	0.03	−4.13 ***
	Self-compassion			0.52	0.03	18.06 ***
Psychological Flourishing	Gender	0.41	196.56 ***	−0.10	0.03	−3.92 ***
	Self-compassion			0.33	0.03	10.71 ***
	Hope			0.40	0.03	13.09 ***

Note: *** *p* < 0.001.

**Table 3:** Test of the moderated mediation model (*N* = 842).

Dependent Variable	Independent Variable	*R* ^2^	*F*	*β*	*SE*	*t*
Hope	Gender	0.34	109.76 ***	−0.01	0.03	−0.29
	Self-compassion			0.43	0.03	14.94 ***
	Emotion regulation			0.31	0.03	10.60 ***
	Self-compassion × Emotion regulation			−0.06	0.02	−2.29 *

Note: * *p* < 0.05, *** *p* < 0.001.

## Data Availability

The data presented in this study are available on request from the corresponding author (the data are not publicly available due to privacy or ethical restrictions).
